# Dynamic synchronization between hippocampal representations and stepping

**DOI:** 10.1038/s41586-023-05928-6

**Published:** 2023-04-12

**Authors:** Abhilasha Joshi, Eric L. Denovellis, Abhijith Mankili, Yagiz Meneksedag, Thomas J. Davidson, Anna K. Gillespie, Jennifer A. Guidera, Demetris Roumis, Loren M. Frank

**Affiliations:** 1 Howard Hughes Medical Institute, University of California, San Francisco, CA USA; 2 Departments of Physiology and Psychiatry, University of California, San Francisco, CA USA; 3 Faculty of Medicine, Hacettepe University, Ankara, Turkey; 4 Kavli Institute for Fundamental Neuroscience, University of California, San Francisco, CA USA

**Keywords:** Spatial memory, Hippocampus

## Abstract

The hippocampus is a mammalian brain structure that expresses spatial representations^1^ and is crucial for navigation^2,3^. Navigation, in turn, intricately depends on locomotion; however, current accounts suggest a dissociation between hippocampal spatial representations and the details of locomotor processes. Specifically, the hippocampus is thought to represent mainly higher-order cognitive and locomotor variables such as position, speed and direction of movement^4–7^, whereas the limb movements that propel the animal can be computed and represented primarily in subcortical circuits, including the spinal cord, brainstem and cerebellum^8–11^. Whether hippocampal representations are actually decoupled from the detailed structure of locomotor processes remains unknown. To address this question, here we simultaneously monitored hippocampal spatial representations and ongoing limb movements underlying locomotion at fast timescales. We found that the forelimb stepping cycle in freely behaving rats is rhythmic and peaks at around 8 Hz during movement, matching the approximately 8 Hz modulation of hippocampal activity and spatial representations during locomotion^12^. We also discovered precisely timed coordination between the time at which the forelimbs touch the ground (‘plant’ times of the stepping cycle) and the hippocampal representation of space. Notably, plant times coincide with hippocampal representations that are closest to the actual position of the nose of the rat, whereas between these plant times, the hippocampal representation progresses towards possible future locations. This synchronization was specifically detectable when rats approached spatial decisions. Together, our results reveal a profound and dynamic coordination on a timescale of tens of milliseconds between central cognitive representations and peripheral motor processes. This coordination engages and disengages rapidly in association with cognitive demands and is well suited to support rapid information exchange between cognitive and sensory–motor circuits.

## Abstract (web summary)

Experiments in rats show that spatial representations in the hippocampus are closely coordinated with the forelimb stepping cycle, particularly when spatial decisions are approaching, and provide insight into how this synchronization supports information processing.

## Main

As animals traverse environments, neural-population representations in the hippocampus often progress through a sequence of spatial positions, including locations behind, at and ahead of the animal’s actual position^13–18^. These sequences repeat at around 8 Hz, concurrent with the theta rhythm^12,19^, and are widely thought to reflect a ‘map’^20,21^ of the available navigational space that informs memory-guided behaviours^4,6^. Consistent with this idea, disrupting hippocampal activity or theta impairs performance in spatial memory tasks^22–24^, in which correct performance involves locomotion to one or more remembered locations. Thus, hippocampal representations can inform decisions^19,25^ that engage locomotor actions. Conversely, locomotor actions move the animal, and hippocampal spatial representations shift to the new position as animals move.

Current accounts posit that hippocampal computations represent a cognitive map or navigational options, but do not posit a link between the timing of these representations and the detailed structure of the locomotor processes (such as the timing of individual footsteps). Specifically, the hippocampus is known to represent higher-order locomotion-related variables, including position, speed and direction^7,26,27^, whereas spinal cord, brainstem and cerebellum circuits represent and drive individual limb movements^8–11^. The coupling of hippocampal representations to limb movements has not been examined, however, and there could be advantages in synchronizing activity across brain systems to facilitate information flow^28^.

**Fig. 1:**
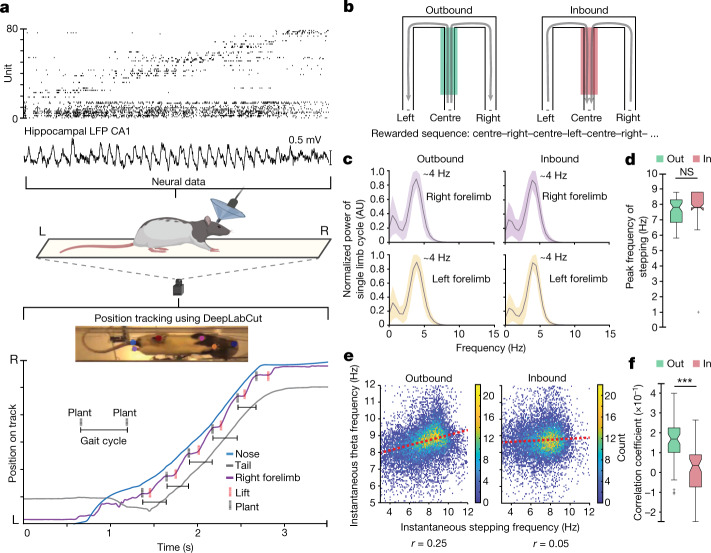
The structure of locomotor activity and its relationship to the hippocampal theta rhythm. **a**, Top, example spike raster from high-density neural recordings of the rat hippocampus (rat 1, *n* = 77 neurons) during navigation on a transparent track. For position tracking, a high-speed camera captures the bottom view at 125 frames per second. A machine-learning algorithm, DeepLabCut (ref. ^65^), is trained to track the nose, forelimbs, hindlimbs and base of the tail of the rat. L, left; R, right; LFP, local field potential. Bottom, simultaneously monitored displacement of the nose, tail, and right forelimb. Plant (black dotted vertical lines) and lift (red dotted vertical lines) times of the right-forelimb stepping cycle are labelled. The schematic of the rat, track and camera was created using Biorender. **b**, Schematic of the w-track task. The behavioural apparatus and rewarded inbound and outbound trajectories are shown with arrows. The centre arm is shaded to denote a region experienced during both inbound and outbound trials and used for quantifications below. **c**, Power spectral density analysis of the stepping cycle of each forelimb during outbound (left) and inbound (right) trials. Trials for all rats combined. Shaded regions represent s.e.m. AU, arbitrary units. **d**, Comparison of the peak frequency of forelimb stepping observed when rats traversed the centre portion of the track during outbound (green) and inbound (red) trials (*n* = 61 epochs in 5 rats, outbound, median: 7.8 Hz, interquartile range (IQR): 6.8–8.3 Hz; inbound, median: 7.8 Hz, IQR: 7.8–8.9 Hz; outbound versus inbound Kruskal–Wallis test: *P* = 0.11; individual animal *P* values: *P* (rat 1), 0.3; *P* (rat 2), 0.1; *P* (rat 3), 0.6; *P* (rat 4), 0.1; *P* (rat 5), 0.2; NS, not significant). Centre lines show the median; box limits indicate the 25th and 75th percentiles; whiskers extend 1.5 × IQR from the 25th and 75th percentiles; outliers are represented by grey symbols. **e**, Correlation between instantaneous forelimb stepping frequency and instantaneous hippocampal theta frequency during outbound (left) and inbound (right) runs, presented in binned scatter plots. The colour scale corresponds to the count in each bin. Trials for all rats combined. **f**, Correlation coefficients between instantaneous forelimb stepping frequency and instantaneous hippocampal theta frequency for outbound and inbound trials across epochs (*n* = 61 epochs in 5 rats, average difference = 0.14, paired two-sided Wilcoxon signed-rank test: *P* = 3.3 × 10^−8^; individual animal *P* values: *P* (rat 1), 0.02; *P* (rat 2), 0.01; *P* (rat 3), 2 × 10^−3^; *P* (rat 4), 0.03; *P* (rat 5), 8 × 10^−3^; adjusted *P* values: *P* (rat 1), 0.02; *P* (rat 2), 0.02; *P* (rat 3), 8 × 10^−3^; *P* (rat 4), 0.03; *P* (rat 5), 0.02). Centre lines show the median; box limits indicate the 25th and 75th percentiles; whiskers extend 1.5 × IQR from the 25th and 75th percentiles; outliers are represented by grey symbols. ***P < 0.0005).

**Extended Data Fig. 1:**
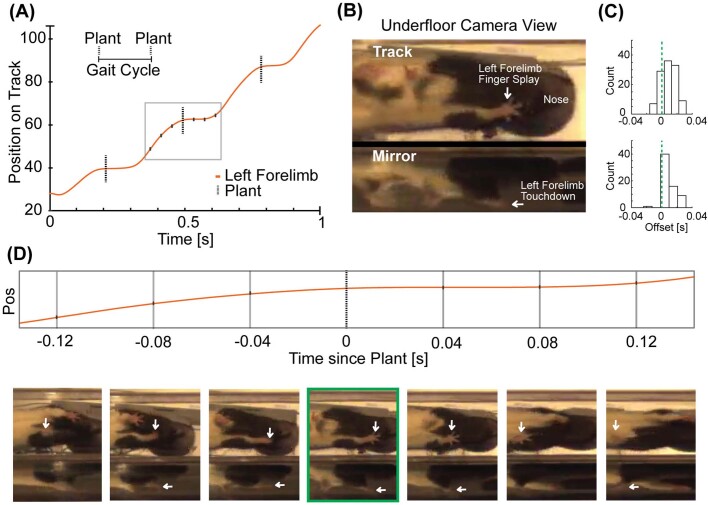
Validation of the locomotor tracking and detecting forelimb plant times. **a**, Example displacement of a reference forelimb and detected plant times (dashed vertical lines) highlighting one complete gait cycle as a rat ran on a transparent track. **b**, Bottom view (track) and a 45-degree mirror view (mirror) are marked to highlight the two views used for manually detecting ‘fingersplay’ and ‘touchdown’ times (see Methods). **c**, Histogram of plant-fingersplay and plant-touchdown offsets shows close correspondence of these two (plant – handsplay median offset/IQR=0.008s, 0.016s, n = 114 plants; plant – touchdown median offset/IQR=0.008s, 0.008s, number of plants = 66). We note here that the human estimates involve somewhat subjective judgements and are not obviously more accurate than those of the algorithm. **d**, Screenshots of three frames on either side of the algorithm detected plant times (highlighted in green box) show the limb placement before and after the plant time.

We therefore simultaneously monitored neural activity in the dorsal hippocampal CA1 region and the stepping rhythm in rats running on transparent behaviour tracks. The resulting data included measurements of the frequency of the theta rhythm and the spiking activity of hippocampal neurons, including spatially selective ‘place’ cells, and a high-resolution undertrack video from which we extracted rats’ limb movements (Fig. 1a, Extended Data Fig. 1 and Supplementary Videos 1 and 2). We focus on data from rats (*n* = 5) learning and performing a hippocampal-dependent spatial memory task on a W-shaped track^29,30^ (Fig. 1b). Running trajectories on this task can be classified into outbound (rat running from the centre well towards either outer well) or inbound (rat running from either outer well towards the centre well), and a correct rewarded sequence corresponds to centre–left–centre–right–centre–left–centre–right, and so on.

## Hippocampal theta and locomotion

Outbound trials require a memory of previous outer-arm choices and are more challenging to learn and perform correctly than inbound trials^29^. In addition, performance on outbound trials is more susceptible to disruption after hippocampal lesions^24^, suggesting that the behaviour of the animal has a higher hippocampal dependence for these trials. We therefore compared the relationship between hippocampal neural variables and stepping cycles across outbound and inbound trials. We restricted these analyses to the centre arm of the track (30–100 cm; see Methods) as rats approached the T-junction where, on outbound trials, they had to choose between the left and right arm.

**Extended Data Fig. 2:**
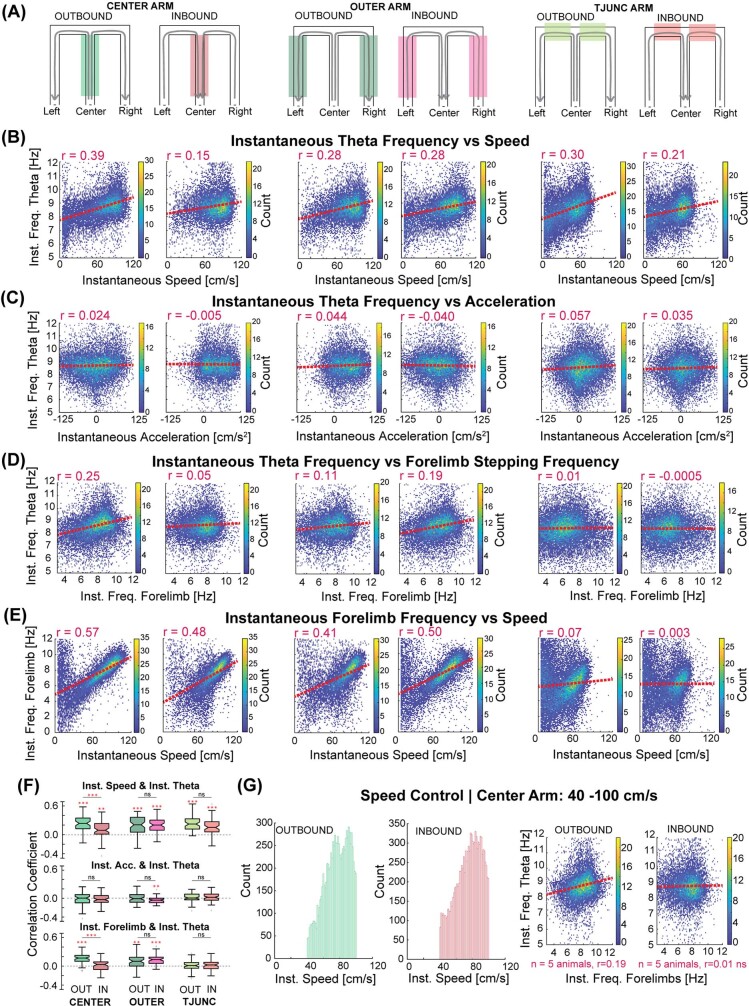
Task-phase-specific relationship between the hippocampal theta rhythm and the stepping rhythm. **a**, Schematic illustrating the different task phases on the w-track. Grey arrows indicate the direction of movement indicating the trial type (outbound or inbound) in different track regions (shaded boxes). This parcellation defined the 6 task phases on the w-track used in this study: Centre Arm – Outbound; Centre Arm – Inbound; Outer Arms – Outbound; Outer Arms – Inbound; T-Junction Arms – Outbound; T-Junction Arms – Inbound. **b**, Density plots of the instantaneous hippocampal theta frequency and instantaneous speed on different task phases on the w-track corresponding to categories in **a**. Correlation coefficients (r) for the combined data are reported on the top left of each panel. Distributions of correlation coefficients computed per epoch for **b**–**e** are shown in **f**. Colour corresponds to the count in each bin. Outbound vs. inbound trials on the centre arm, average correlation difference = 0.14, Kruskal–Wallis test: p = 7.4 x 10^−6^; individual animal p values: p (rat 1): 7 x 10^−3^; p (rat 2): 0.5, p (rat 3): 3 x 10^−4^, p (rat 4): 0.07, p (rat 5): 9 x 10^−3^; Benjamini–Hochberg adjusted p values: p (rat 1): 0.01, p (rat 2): 0.5, p (rat 3): 1 x 10^−3^, p (rat 4): 0.09, p (rat 5): 0.01 (comparison to no correlation: outbound, t-test: p = 7.8 x 10^−18^, individual animal p values: p (rat 1): 9 x 10^−4^, p (rat 2): 1 x 10^−4^, p (rat 3): 6 x 10^−5^, p (rat 4): 9 x 10^−5^, p (rat 5): 1 x 10^−4^; Benjamini–Hochberg adjusted p values: p (rat 1): 9 x 10^−4^, p (rat 2, rat 3, rat 4, rat 5): 2 x 10^−4^; inbound, t-test: p = 6.2 x 10^−5^, individual animal p values: p (rat 1): 0.1, p (rat 2): 1 x 10^−5^, p (rat 3): 0.2, p (rat 4): 4 x 10^−3^, p (rat 5): 7 x 10^−3^; Benjamini–Hochberg adjusted p values: p (rat 1): 0.1, p (rat 2): 1 x 10^−4^, p (rat 3): 0.2, p (rat 4): 9 x 10^−3^, p (rat 5): 0.01). **c**, Density plots of the instantaneous hippocampal theta frequency and instantaneous acceleration of the rat on different task phases on the w-track show low correlation coefficients (5 rats, 61 epochs). These variables were not consistently modulated across rats as evidenced by the distribution of correlation coefficients (r, Extended Data Fig. 1f) on the centre arm during outbound and inbound trials (median correlation outbound: 3 x 10^−3^; t-test for outbound values compared to 0, p = 0.41; median correlation inbound: −0.03; t-test for inbound values compared to 0, p = 0.42). Colour corresponds to the count in each bin. **d**, Density plots of the instantaneous hippocampal theta frequency and instantaneous forelimb stepping frequency on different task phases on the w-track (5 rats, 61 epochs). Forelimb stepping frequency was strongly correlated with hippocampal theta frequency during outbound trials on the centre arm (two-sided t-test of r values compared to 0: p = 4.8 x 10^−16^; individual animal p values: p (rat 1): 5 x 10^−4^, p (rat 2): 6 x 10^−4^, p (rat 3): 1 x 10^−4^, p (rat 4): 1 x 10^−3^, p (rat 5): 1 x 10^−4^; Benjamini–Hochberg method adjusted p values: p (rat 1): 7 x 10^−4^, p (rat 2): 7 x 10^−4^, p (rat 3): 3 x 10^−4^, p (rat 4): 1 x 10^−3^, p (rat 5): 3 x 10^−4^) but we found no consistent correlation on inbound runs (two-sided t-test of r values compared to 0: p = 0.25; individual animal p values: p (rat 1): 0.1, p (rat 2): 0.1, p (rat 3): 0.3, p (rat 4): 0.8, p (rat 5): 0.02; Benjamini–Hochberg adjusted p values: p (rat 1): 0.2, p (rat 2): 0.2, p (rat 3): 0.3, p (rat 4): 0.8, p (rat 5): 0.1). Further, the outbound correlation coefficients were significantly different from those observed during inbound trials on the centre arm (Fig. 1d). Distribution of correlation coefficients for other task phases calculated per epoch is reported in **f**. Colour corresponds to the count in each bin. Note, Extended Data Fig. 1 row D, column 1 is the same as Fig. 1e. Centre lines show the medians; box limits indicate the 25th and 75th percentile; whiskers extend 1.5 times the interquartile range from the 25th and 75th percentiles; outliers are represented by grey symbols. **e**, Density plots of the instantaneous forelimb stepping frequency and instantaneous running speed of the rat on different task phases on the w-track (n = 61 epochs in 5 rats). Colour corresponds to the count in each bin. **f**, Distribution of the correlation coefficients computed per epoch during different task phases on the w-track. Asterisks (*) indicate that the distribution of correlation coefficients is significantly different from zero (two-sided t-test, p < 0.05). Comparisons of the same track region experienced during outbound and inbound portions on the w-track are highlighted using paired two-sided Wilcoxon signed-rank test. *p < 0.05, **p < 0.005, ***p < 0.0005. **g**, Running speed control: correlations controlled for the rat’s running speed on the centre region of the track during outbound and inbound trials. Analysis was restricted to running speeds of 40–100 cm/s. Histogram of instantaneous speeds during outbound and inbound trials included for analysis (left) and resulting binned scatter plots (right) show that outbound trials on the centre arm have higher correlation coefficients compared to those on inbound portions of the centre arm.

We first examined the well-known correlation between running speed and the frequency of the theta rhythm^27,31^ in that part of the track (*n* = 61 total recording epochs across 5 rats). Notably, we found that this correlation was stronger on outbound trials than inbound trials (5 rats, 61 epochs, outbound versus inbound, average difference = 0.14, Kruskal–Wallis test: *P* = 7.4 × 10^−6^; see figure legends for individual animal *P* values; Extended Data Fig. 2a,b,f). We note that we, like others^32^, did not observe a consistent significant correlation between running speed and acceleration on either outbound or inbound trials, in contrast to another previous report^33^ (Extended Data Fig. 2c,f).

**Extended Data Fig. 3:**
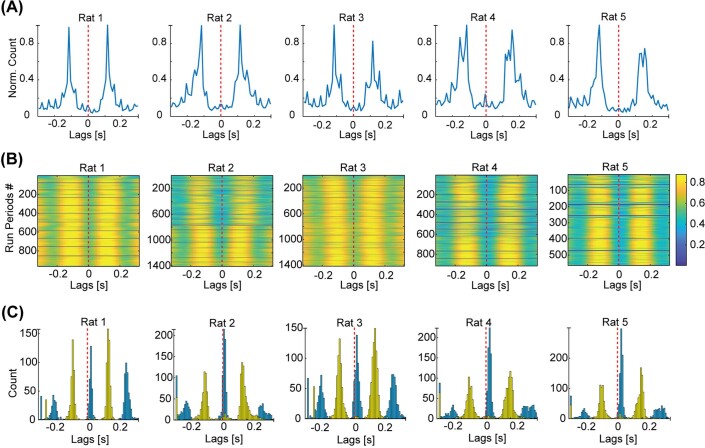
Individual forelimb cross-correlations during runs. **a**, Normalized cross-correlograms of left- and right-forelimb plant times on the w-track show a trough at zero indicating that the dominant gait motif in the reported running speeds corresponds to trotting, where the limbs strike the ground in alternating sequence. **b**, Cross-correlation heat maps of individual left and right-forelimb stepping cycles during each run on the track. **c**, The distribution of maxima (peaks) and minima (troughs) values of the cross-correlations of each run shown in **b** is plotted in a histogram to display a lack of overlap between the left and right forelimb, confirming that the rats are rarely if ever using a gait where both forelimbs hit the ground at the same time (for example, during bounding).

The differential coupling of movement speed and theta frequency as a function of trial type led us to wonder whether the detailed structure of locomotor processes might also be dynamically coupled to hippocampal theta rhythm—a possibility that has been raised in previous work^34,35^. As locomotion consists of cyclic movements of the limbs, we first asked how the overall frequency of these movements compared to the frequency of theta. We found that as the rats traversed the centre arm, each forelimb rhythmically moved at a peak frequency of around 4 Hz, together propelling the rats at a stepping frequency of around 8 Hz (Fig. 1c and Extended Data Fig. 3). This peak frequency matched the approximately 8-Hz peak frequency of the theta rhythm and was not different between inbound and outbound trials (5 rats, 61 epochs, average difference = −0.26 Hz, Kruskal–Wallis test: *P* = 0.11; Fig. 1d). This highly consistent peak frequency contrasted with previous results in head-fixed mice, in which a wider range of stepping frequencies was reported^34^, suggesting that head-fixation might introduce additional locomotor variability. Next, we directly assessed whether theta frequency was related to the instantaneous frequency of forelimb stepping and whether this relationship varied by trial type.

Here, again, we found a trial-type-specific coupling. There was a consistent positive correlation between theta and forelimb stepping frequencies on outbound runs (5 rats, 61 epochs, *t*-test of *r* values compared to 0: *P* = 4.8 × 10^−16^; Fig. 1e and Extended Data Fig. 2f), but no consistent correlation on inbound runs (5 rats, 61 epochs, *t*-test of *r* values compared to 0: *P* = 0.25; Extended Data Fig. 2f). Furthermore, the outbound correlations were significantly larger than the inbound correlations (5 rats, 61 epochs, average difference = 0.14, Wilcoxon signed-rank test: *P* = 3.3 × 10^−8^; Fig. 1f). These relationships could not be explained by differences in running speed (Extended Data Fig. 2g). Combined, these results showed that the theta rhythm was more closely coupled with movement speed and forelimb stepping frequency specifically during the more difficult outbound trials.

## Synchronization in outbound trials

We then asked whether there was also a relationship between stepping and the hippocampal representation of space. Outbound runs on the centre arm of the w-track are known to strongly engage theta-paced representations that typically progress, on each cycle, from locations closer to the animal’s actual position toward possible future locations^6,12–19^, allowing us to ask whether this progression from current to future is synchronized with stepping.

We used a clusterless decoding algorithm^36^ to determine the location represented by hippocampal spiking activity at high temporal resolution (2-ms time bins; see Methods). We then calculated the offset between that estimate of ‘mental position’ and the actual position of the nose of the rat (see Methods) to create a distance metric (hereafter, ‘decode-to-animal distance’) that captures the deviation between represented and actual position^34^. We focused on the centre region of the track (60–100 cm) on outbound trials, as that region corresponds to the rat approaching the navigational choice point.

We then asked whether the decode-to-animal distance was related to the stepping cycle. The high-resolution pose estimation used in this study enabled us to estimate the times when the rat’s forelimbs first touched the track on each cycle (plant times; see Methods). To measure the relationship with ongoing steps, we used these plant times because these are distinct and identifiable reference points in the stepping cycle and correspond to periods of maximum cutaneous and proprioceptive input from the limbs to the central nervous system^37,38^. Here we limited our analyses to those epochs and times in which we could reliably decode the hippocampal representation (see Methods).

**Fig. 2:**
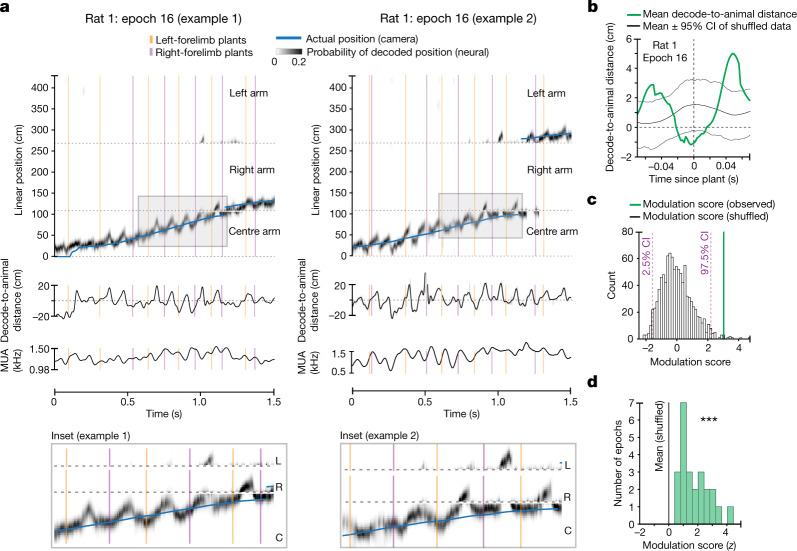
Synchronization between hippocampal spatial representations and forelimb plant times. **a**, Estimation of the represented position on the basis of clusterless decoding during outbound runs on the centre arm of the w-track. Blue trace represents the linearized position of the rat’s nose. Grey density represents the decoded position of the rat on the basis of spiking. Note that the decoded position can be ahead of, near or behind the rat’s current position. Orange and purple vertical lines represent the plant times of the left and the right forelimb, respectively. Shaded box indicates inset enlarged below. C, centre; R, right; L, left. **b**, Mean decode-to-animal distance trace triggered by forelimb plant times that precede non-local representations greater than 10 cm ahead of the rat’s current position for the selected region (60–100 cm) (green line; data from rat 1, epoch 16). Grey lines represent the 95% confidence interval (CI) of the shuffled distribution. The dotted line at zero indicates decode-to-animal distance values corresponding to the actual position of the rat’s nose, and positive or negative values indicate represented positions ahead or behind the actual position of the rat, respectively. **c**, Decode-to-animal distance modulation score of the observed data (vertical line, green) and the histogram of the modulation score for the shuffled distributions (bars, grey). **d**, Distribution of the decode-to-animal distance modulation score for the observed data in all rats (green bars) versus the mean of the modulation score for the shuffled data (black vertical line; *n* = 24 epochs in 4 rats, two-sided *t*-test: *P* = 1.6 × 10^−8^; individual animal *P* values: *P* (rat 1), 4 × 10^−3^; *P* (rat 2), 5 × 10^−3^; *P* (rat 3), 4 × 10^−4^; *P* (rat 5), 0.04; Benjamini–Hochberg adjusted *P* values: *P* (rat 1), 7 × 10^−3^; *P* (rat 2), 7 × 10^−3^; *P* (rat 3), 2 × 10^−3^; *P* (rat 5), 0.04). *P* < 0.0005.

**Extended Data Fig. 4:**
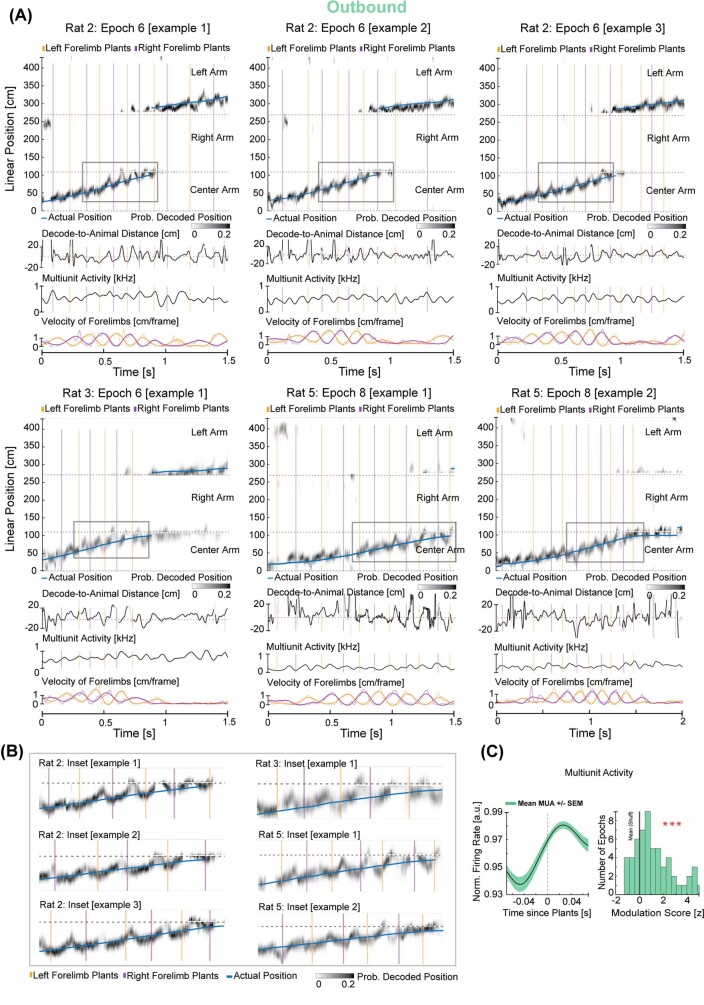
Prominent synchronization of forelimb plant times with neural representation of current position during outbound trials. **a**, Individual examples as in Fig. 2 from rat 2, rat 3 and rat 5. Blue trace represents the linearized position of the rat’s nose. Grey density represents the decoded position of the rat based on spiking. Note that the decoded position can be ahead of, near, or behind the rat’s actual position. Orange and purple vertical lines represent the plant times of the left and right forelimb, respectively. **b**, Insets correspond to shaded areas in **a** enlarged to highlight individual examples of the synchronization between hippocampal representations and forelimb plants. Note, forelimb plant times coincide with hippocampal representation of the actual location of the rat. **c**, Left, forelimb-plant-triggered MUA (mean +/− SEM) is modulated during outbound trials. Right, correspondingly, the distribution of the MUA modulation score for the observed data in all rats (green, bars) is significantly different from the mean of the modulation score for the shuffled data (black, vertical line; n = 61 epochs in 5 rats, two-sided t-test: p = 3.9 x 10^−7^; individual animal p values: p (rat 1): 4 x 10^−4^, p (rat 2): 0.07, p (rat 3): 0.5, p (rat 4): 6 x 10^−4^, p (rat 5): 3 x 10^−3^; adjusted p values: p (rat 1): 1 x 10^−3^, p (rat 2): 0.09, p (rat 3): 0.5, p (rat 4): 1 x 10^−3^, p (rat 5): 5 x 10^−3^, consistent trends observed in 4/5 rats). Note, rat 3 had 15 electrodes targeted in the hippocampus instead of 30 for rat 1, rat 2, rat 4 & rat 5. ***p < 0.0005.

We found that the plant times of the left and right forelimbs corresponded to hippocampal representations of position close to the actual location of the rat (Fig. 2a–c and Extended Data Fig. 4a,b). In between these plant times, the hippocampal representation of position typically progressed towards possible future locations and then reset to the actual position of the rat in conjunction with the next forelimb plant (Supplementary Video 3). To quantify this relationship, we focused on theta sequences with an appreciable representation of future locations^39^ (that is, more than 10 cm ahead of the actual location of the rat’s nose; see Methods) and computed an epoch-wise decode-to-animal distance modulation score (Fig. 2c; see Methods) that captured the consistency of the synchronization between the hippocampal representations and forelimb plant times. We note that the epoch-wise average of the decode-to-animal distance trace (Fig. 2b) shows smaller values than 10 cm owing to variability in the temporal offset of the time of crossing beyond 10 cm across multiple plants. The measured distribution of epoch-wise modulation scores was greater than the modulation computed from a series of shuffled datasets in which the plant times on each trial were shifted by a value chosen from a uniform distribution spanning ±70 ms (4 rats, 24 epochs, observed modulation versus mean of shuffles for each epoch, 60–100 cm on w-track: *t*-test: *P* = 1.6 × 10^−8^; Fig. 2d).

This synchronization between plant times and theta sequences also manifested as a synchronization between plant times and the overall multiunit activity (MUA) levels in the hippocampus. In conjunction with rhythmic theta sequences, hippocampal neurons fire rhythmically, such that multiunit firing rates wax and wane on each theta cycle^13^. We computed the degree of modulation of MUA relative to plant times for each epoch and compared it with the mean of the modulation scores for the shuffled distributions (see Methods). As expected from the relationship between plant times and theta sequences, there was a highly significant temporal modulation of MUA and plant times (5 rats, 61 epochs, 60–100 cm on w-track: *t*-test: *P* = 3.9 × 10^−7^; Extended Data Fig. 4c).

We also analysed whether the prospective position represented after a left- or right-forelimb plant coincides with a left or right representation of space^6^ as the rat approaches the choice point (see Methods). We did not observe a consistent organization in our data (4 rats, 24 epochs, Kruskal–Wallis test: *P* = 0.24; individual animal *P* values: *P* (rat 1), 0.7; *P* (rat 2), 0.2; *P* (rat 3), 0.6; *P* (rat 5), 0.2). These results are consistent with previous work on the w-track^6^ that suggested that the left–right neural representations do not reflect the eventual choice of the animal and are instead consistent with navigational options available to downstream cortical and subcortical regions involved in action selection.

## Dynamic coupling of space and steps

**Fig. 3:**
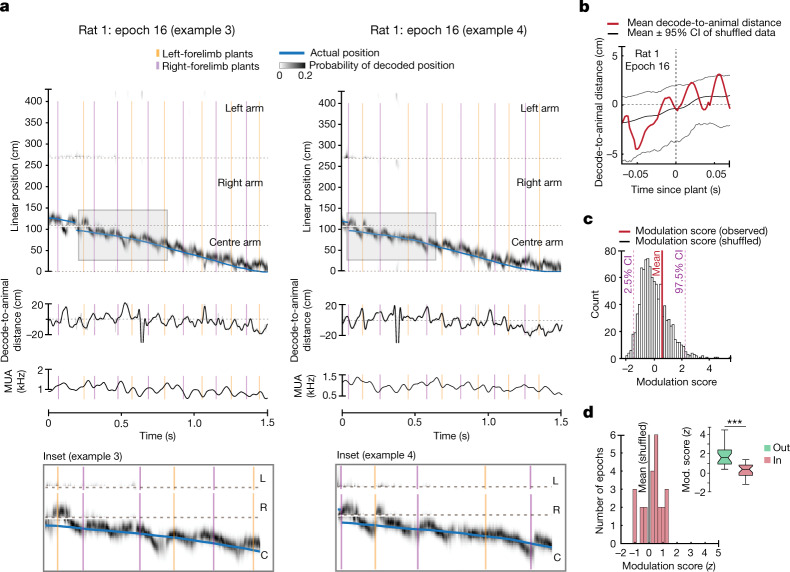
Engagement between hippocampal neural representations and stepping rhythm is dependent on task phase. **a**, Estimation of the represented position on the basis of clusterless decoding (as in Fig. 2) during inbound runs on the centre arm of the w-track. Blue trace represents the linearized position of the rat’s nose. Grey density represents the decoded position of the rat on the basis of spiking. Orange and purple vertical lines represent the plant times of the left and the right forelimb, respectively. Note that the decode-to-animal distance and MUA rhythmically fluctuate during the inbound runs. Shaded box indicates inset enlarged below. C, centre; R, right; L, left. **b**, Decode-to-animal distance trace triggered by forelimb plant times that precede non-local representations greater than 10 cm ahead of the rat’s current position for the selected region (60–100 cm) (red line; data from rat 1, epoch 16). Grey lines represent the 95% CI of the shuffled distribution. The dotted line at 0 indicates decode-to-animal distance values corresponding to the actual position of the rat’s nose. **c**, Decode-to-animal distance modulation score of the observed data (vertical line, red) and the histogram of the modulation score for the shuffled distributions (bars, grey). **d**, Distribution of modulation scores for the observed data in all rats (red bars) and the mean of the modulation score for the shuffled data (grey vertical line, *n* = 24 epochs in 4 rats, two-sided *t*-test: *P* = 0.08; individual animal *P* values: *P* (rat 1), 0.8; *P* (rat 2), 0.4; *P* (rat 3), 0.3; *P* (rat 5), 0.01; adjusted *P* values: *P* (rat 1), 0.8; *P* (rat 2), 0.5; *P* (rat 3), 0.5; *P* (rat 5), 0.05). Inset, comparison between the decode-to-animal distance modulation score during outbound (green) and inbound (red) runs on the w-track shows a stronger modulation of decode-to-animal distance by forelimb plants during outbound runs on the centre arm (4 rats, 24 epochs, Wilcoxon signed-rank test: *P* = 4.3 × 10^−5^; individual animal *P* values: *P* (rat 1), 0.03; *P* (rat 2), 0.04; *P* (rat 3), 0.03; *P* (rat 5), 0.06; adjusted *P* values: *P* (rat 1), 0.05; *P* (rat 2), 0.05; *P* (rat 3), 0.05; *P* (rat 5), 0.06; ***P < 0.0005). Centre lines show the median; box limits indicate the 25th and 75th percentiles; whiskers extend 1.5 × IQR from the 25th and 75th percentiles.

**Extended Data Fig. 5:**
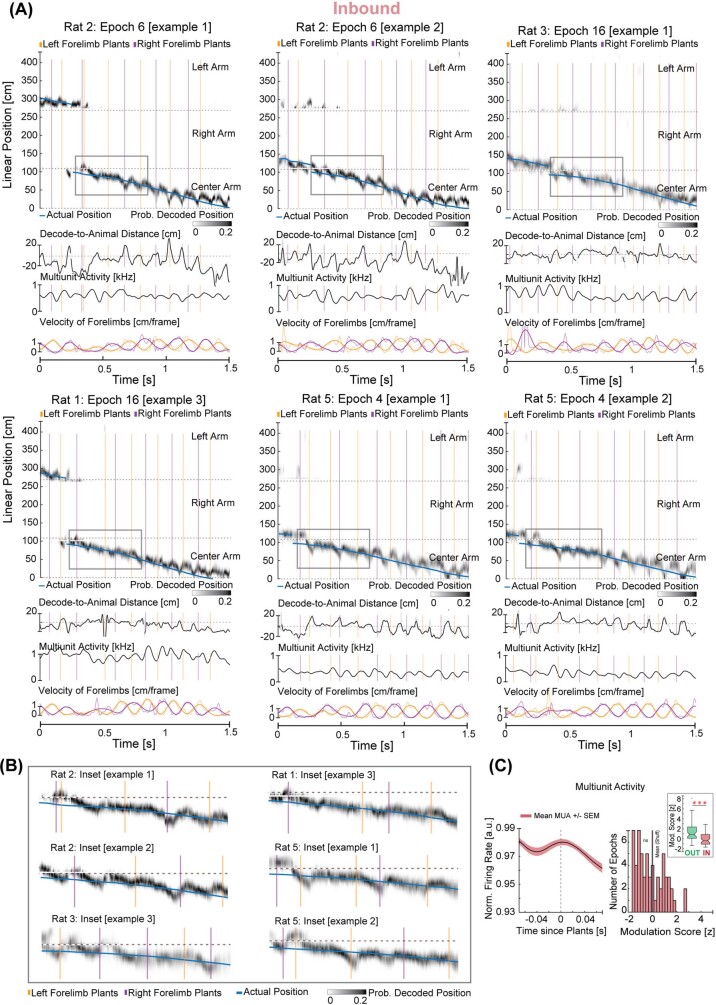
Examples of hippocampal spatial representations and forelimbs during inbound trials. **a**, Individual examples as in Fig. 2 from rat 2, rat 3 and rat 5. Blue trace represents the linearized position of the rat’s nose. Grey density represents the decoded position of the rat based on spiking. Orange and purple vertical lines represent the plant times of the left and right forelimb, respectively. Note that the decode-to-animal distance, MUA, and stepping rhythmically fluctuate during the inbound runs—shaded regions in zoomed insets below. **b**, Insets are shaded areas in **a** enlarged to highlight individual examples of hippocampal representations and forelimb plants during inbound trials on the centre arm. Note, the lack of coordination between forelimb plants and hippocampal representation during inbound trials. On these trials, forelimb plants could occur when hippocampal decode represents positions that are ahead, concurrent, or behind the actual location of the rat. **c**, Left, forelimb-plant-triggered MUA (mean +/− SEM) shows low modulation during inbound trials. Right, correspondingly, the distribution of the MUA modulation score for the observed data in all rats (red, bars) is significantly different from the mean of the modulation score for the shuffled data (black, vertical line, 5 rats, 61 epochs, two-sided t-test: p = 0.37, individual animal p values: p (rat 1): 0.6, p (rat 2): 0.1, p (rat 3): 0.3, p (rat 4): 0.8, p (rat 5): 0.2). Inset: comparison of the MUA modulation score during the outbound (green) and inbound (red) runs on the centre arm of the w-track shows a more robust modulation during the outbound portions (n = 61 epochs in 5 rats, paired two-sided Wilcoxon signed-rank test: p = 1.7 x 10^−7^, individual animal p values: p (rat 1): 2 x 10^−3^, p (rat 2): 9 x 10^−3^, p (rat 3): 0.1, p (rat 4): 5 x 10^−3^, p (rat 5): 0.03; adjusted p values: p (rat 1): 0.01, p (rat 2): 0.01, p (rat 3): 0.1, p (rat 4): 0.01, p (rat 5): 0.04). ***p < 0.0005. Centre lines show the medians; box limits indicate the 25th and 75th percentile; whiskers extend 1.5 times the interquartile range from the 25th and 75th percentiles; outliers are represented by grey symbols.

If the coordination between locomotor processes and hippocampal representations is specifically engaged at times of higher cognitive load, we would expect this relationship to be prevalent on outbound trials but not on inbound trials. We therefore examined this synchronization during the inbound runs on the centre arm of the w-track (Fig. 3a and Extended Data Fig. 5a,b).

**Extended Data Fig. 6:**
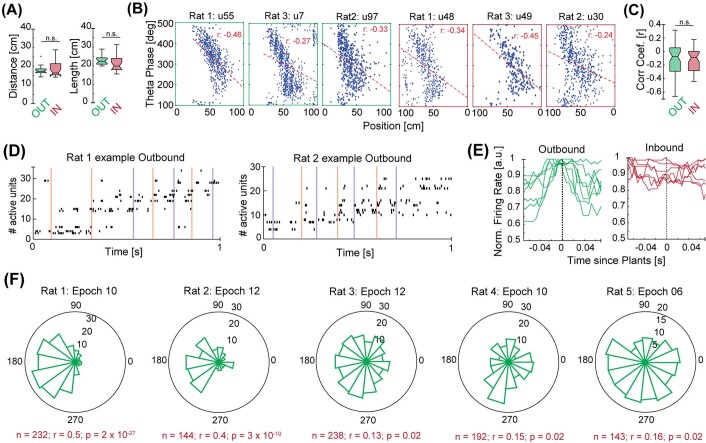
Theta sequences and theta-phase precession are prevalent during both outbound and inbound task phases. **a**, Left, distribution of peaks (median per epoch) of the decode-to-animal distance trace on outbound and inbound task phases on the centre arm were not statistically different (n = 24 epochs in 4 rats; outbound median: 17 cm, inbound median: 16 cm inbound; Kruskal–Wallis test: p = 0.39; individual animal p values: p (rat 1): 0.7, p (rat 2): 0.5, p (rat 3): 0.6, p (rat 5): 0.8). Right, in a complementary approach, we parsed the decode-to-animal-distance trace by theta troughs and compared their length (median per epoch) during outbound and inbound portions on the track. Here again, we did not find a consistent difference between inbound and outbound task phases on the centre arm (median length outbound: 22 cm, median length inbound 19 cm, p: 0.08; individual animal p values: p (rat 1): 3 x 10^−3^; p (rat 2): 0.9, p (rat 3): 0.9, p (rat 5): 0.8). **b**, Example phase-precession plots of three putative pyramidal cells during the outbound (green outer boxes) and inbound (red outer boxes) task phases on the centre arm of the track. Correlation coefficients (r, red text). **c**, Box plots showing the distribution of correlation coefficients computed for each active putative pyramidal cell in 3 epochs across each of 3 rats (Kruskal–Wallis test p = 0.42, individual animal p values: p (rat 1): 0.8, p (rat 2): 0.3, p (rat 3): 0.4; number of cells outbound: 57; number of cells inbound: 46). Centre lines show the medians; box limits indicate the 25th and 75th percentile; whiskers extend 1.5 times the interquartile range from the 25th and 75th percentiles. **d**, Examples of spike rasters of active putative pyramidal cells during outbound runs starting on the centre arm of the w-track. Cells are ordered by the mean of spike times on the outbound runs on the track. The plots illustrate that theta sequences can be observed in the spiking activity of pyramidal cells even when putative interneurons are excluded. Coloured vertical lines are plant times of the right (purple) and left (orange) forelimbs. **e**, Examples of forelimb-plant-triggered activity of putative pyramidal cells (each line corresponds to one cell, n = 6 examples from Rat 1 epoch 16) active during outbound and inbound task phases on the centre arm of the track, respectively. Left, cells active during outbound runs (green). Right, cells active during inbound runs (red). Note the modulation of spiking activity by steps is also observed at the level of individual pyramidal neurons. These results are complementary to Extended Data Fig. 4c and Extended Data Fig. 5c. **f**, Examples of circular histograms showing the prominent phase relationship between forelimb plants and hippocampal theta oscillations. Bin size: 24 degrees.

Although we observed clear theta sequences on the inbound runs, we did not observe significant modulation of spatial representations relative to plant times during these periods. This was evident in individual examples (Fig. 3b) and in the distribution of the measured decode-to-animal distance modulation scores, which was not consistently different from the respective shuffled distributions (4 rats, 24 epochs, *t*-test: *P* = 0.08; Fig. 3c,d). These inbound scores were significantly smaller than those observed during outbound runs, indicating a low degree of synchronization between the plant times and decode-to-animal distance trace during inbound trials (4 rats, 24 epochs, mean modulation score outbound: 1.75; mean modulation score inbound: 0.27; Wilcoxon signed-rank test: *P* = 4.3 × 10^−5^; Fig. 3e). These differences could not be explained by a difference in the structure of theta sequences or single-cell phase precession between outbound and inbound task phases (Extended Data Fig. 6). Similarly, we did not find a significant modulation of hippocampal MUA by forelimb plant times during inbound runs (Extended Data Figs. 5c and 6), and the MUA modulation scores were significantly smaller for inbound than for outbound runs (5 rats, 61 epochs, mean modulation score outbound: 1.48; mean modulation score inbound: −0.13, Wilcoxon signed-rank test: *P* = 1.7 × 10^−7^; Extended Data Fig. 5c). Thus, our data indicate that stepping and hippocampal neural variables are synchronized dynamically according to task phase.

**Extended Data Fig. 7:**
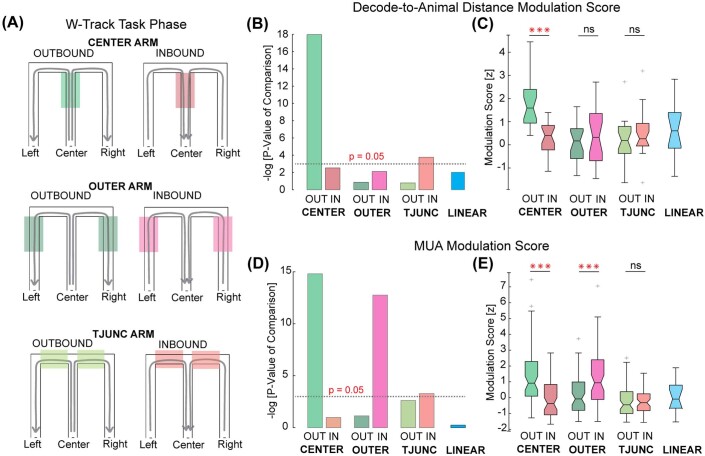
Modulation of decode-to-animal distance and MUA by forelimb plant times is most prominent during outbound trials on the centre arm of the w-track. **a**, Schematic illustrating the different task phases on the w-track. Shaded portions are highlighted to illustrate regions on the track included for the analysis. **b**, Comparison of the decode-to-animal distance modulation score on different portions of the w-track and a separate linear track. The negative log of the p-value corresponds to the comparison of the modulation score on each portion of the track to that of its shuffled distributions. The dotted line corresponds to p = 0.05 (t-test). Note that while there was a statistically significant decode-to-animal-distance modulation on the T-junction arm during inbound trials when all rats were combined, this was not significant in any individual rats (two-sided t-test, p = 0.02; individual animal p values: p (rat 1): 0.6, p (rat 2): 0.1, p (rat 3): 0.6, p (rat 5): 0.1). **c**, Box plots show the distribution of decode-to-animal distance modulation scores calculated per epoch on different portions of the w-track and linear track. Asterisks (*) indicate that the paired comparisons between outbound and inbound trial types on the same track region were significant (Kruskal–Wallis test; p = 0.05). ***p < 0.0005. Centre lines show the medians; box limits indicate the 25th and 75th percentile; whiskers extend 1.5 times the interquartile range from the 25th and 75th percentiles; outliers are represented by grey symbols. **d**, Same as **b**, but for MUA modulation score. Note, as in the case of decode-to-animal-distance modulation, a statistically significant MUA modulation was observed on inbound trials on the T-junction arms (two-sided t-test, p = 0.04; individual animal p values: p (rat 1): 0.2, p (rat 2): 0.5, p (rat 3): 0.2, p (rat 4): 0.7, p (rat 5): 0.2). **e**. Same as **c**, but for MUA modulation score. ***p < 0.0005. Centre lines show the medians; box limits indicate the 25th and 75th percentile; whiskers extend 1.5 times the interquartile range from the 25th and 75th percentiles; outliers are represented by grey symbols.

We then expanded these analyses to other regions of the track, including the outer arms and the regions just past the T-junction (Extended Data Fig. 7a). We reasoned that if the presence of a difficult upcoming choice modulated the synchronization between hippocampal spatial representations and locomotor processes, we would see clear evidence for synchronization on outbound trials before the choice point and little evidence for synchronization past the choice point. Conversely, on inbound trials, we might find evidence for synchronization in the outer arms or T-junction regions, but a lack of evidence for synchronization in the centre arm.

Our results were consistent with those conjectures. We compared the decode-to-animal distance across track regions and found that the most robust modulation was observed during outbound runs on the centre arm (Extended Data Fig. 7b). We also observed strong modulation of MUA at these times (Extended Data Fig. 7d). Furthermore, we found some evidence for decode-to-animal distance and MUA modulation on inbound runs on the T-junction arm—locations that also preceded a choice (decode-to-animal distance: *t*-test, *P* = 0.02; MUA: *t*-test, *P* = 0.04; Extended Data Fig. 7b–d).

## Discussion

Our results reveal a notable synchronization between ongoing hippocampal spatial representations and the stepping cycle as animals approach upcoming spatial decisions. Previous work showed that various physiological rhythms (such as breathing, head-scanning, saccades and so on) could be coupled to hippocampal theta rhythms^34,35,40–44^; our findings demonstrate coupling of ongoing steps not only to hippocampal theta but also to MUA and the microstructure of spatial representations. This coupling is strongest as animals approach a decision point, and synchronizes the rhythms such that the hippocampal representation returns to a location close to the animal’s actual position at the time at which the forelimbs strike the ground.

This dynamic relationship is unlikely to reflect a direct drive from sensory inputs to the hippocampus or from the hippocampus to motor outputs. Specifically, the representation of space typically returned to a position close to the actual location of the animal before plant times (Fig. 2 and Extended Data Fig. 4), and there is no evidence for a direct hippocampal output to motor effectors. This relationship is also unlikely to reflect a dominant synchronizing drive from another sensory–motor system. Previous studies have documented dynamic coupling between hippocampal theta and the respiration and whisking rhythms^35,43^, which are themselves strongly coupled. However, this coupling is typically seen at frequencies outside the 7–9 Hz range^45–47^, in which stepping and theta are strongly synchronized. Furthermore, although theta and stepping frequencies both increase roughly linearly with speed (Fig. 1e), that is not the case for respiration and whisking^48^. Thus, the known properties of coupling between respiration or whisking rhythms and the hippocampus are not obviously consistent with these rhythms having a dominant role in driving the synchronization we observe.

We instead propose that the precise and dynamic coupling between stepping and hippocampal activity reflects a distributed mechanism that coordinates internal hippocampal representations about space (which rhythmically sweep into the future and then return to the animal’s location at theta timescales during behaviour) with ongoing locomotor processes (which provide the strongest sensory signals when the limb strikes the ground^37,38^), such that they concurrently reflect information about the actual position of the animal during plant times. Of note, in between consecutive plant times, the hippocampus often represents potential future trajectories. Such an organization^28^ is well suited to segregating information related to environmental sampling^49^ versus planning potential future trajectories^6,50^ across brain regions involved in decision-making on fast timescales^51–53^. Conversely, a lack of synchronization—as in inbound trials in the centre arm—may reflect a relative lack of engagement of hippocampal representations in guiding ongoing behaviour at these times^29^.

Our data also raise the possibility that previous results with regard to rhythmic medial entorhinal cortical neuronal coupling with speed^54,55^ (based on analyses of autocorrelograms at different running speeds) could reflect coupling between the rhythmic coding of location and the stepping cycle. In addition, our data complement work showing that a large proportion of the variance observed in neocortical activity during routine behaviours and decision-making tasks is related to movement^56,57^. However, although those reports identified static relationships on timescales of around 2–5 s, we found that locomotor processes are dynamically synchronized with ongoing cognitive representations in the hippocampus on timescales of tens of milliseconds. The existence of these precisely timed representations in the hippocampus—a structure anatomically distant from the sensory–motor periphery—demonstrates widespread coupling between movements, associated sensory inputs and higher-order cognitive representations.

This dynamic coupling may also exist across species. There is evidence for synchronization between saccades and the hippocampal theta rhythm in non-human primates^40^ and a relationship between button presses and hippocampal theta frequency coherence in humans^58^. Our findings raise the possibility of synchronization between hippocampal representations and movement across species, and further suggest that this synchronization would be engaged specifically at times at which hippocampal representations are important for storing memories or guiding behaviour.

In the context of evolution, the ‘bauplan’ of locomotion and its coordination with spinal and cortical circuits has been conserved over the course of evolutionary history, with marked similarities between present-day mammals and lampreys^59,60^, despite differences in major mammalian locomotor modes (for example, flying, quadrupedal or bipedal motion). Moreover, naturalistic behaviours are accompanied by a complex interaction between multiple sensory–motor processes, such as breathing, whisking, visual flow, stepping and so on, each with its own characteristic frequencies in a given species. Hippocampal representations of space have also been reported across multiple species^61–64^ but there are also known cross-species differences in the rhythmicity and power of theta oscillations (for example, some animals are reported to have theta only in bouts). We speculate that there could be coupling between spatial representations and sensory–motor processes across species, but that the specific nature of this coupling would depend on species-specific sensory–motor and cognitive infrastructure.

## Methods

### Experimental model and animals

Neural activity (cellular firing and local field potential) was recorded from the CA1 region of the dorsal hippocampus in five male Long-Evans rats (*Rattus norvegicus*; 5–9 months old, weighing 500–650 g) performing a spatial alternation w-track task^6,29^. Rats were housed in a humidity- and temperature-controlled facility with a 12-h light–dark cycle. Rats were housed with littermates before experimental manipulation and singly housed in enriched cages during training and food-restriction protocols. All experimental procedures were in accordance with the University of California San Francisco Institutional Animal Care and Use Committee and US National Institutes of Health guidelines.

### Behavioural task and neural recordings

Rats were deprived of food to 85% of their baseline weight and pretrained to run on a linear track for liquid reward (sweetened evaporated milk). This training was done to familiarize the rats with reward wells. After the rats alternated between the two reward wells reliably, they were put back on complete food for at least one week before the implantation surgery. During the surgery, rats were implanted with microdrives^66^ containing 30 (3 rats), 24 (1 rat) or 16 (1 rat) independently movable 4-wire electrodes targeting the CA1 region of the dorsal hippocampus (all rats), polymer probes in frontal cortical areas (1 rat) and an optic fibre in the medial septum (1 rat). Only hippocampal data were analysed in this study. The hippocampal target electrodes were slowly advanced towards the pyramidal cell layer over two to three weeks. Before running on the w-track task (100 cm × 100 cm; track width 10 cm), four rats also ran on other dynamic foraging tasks in different rooms or contexts. The data presented in this paper are from eight to twenty 15–20-min run sessions during learning and performance on the w-track task (number of epochs per rat: rat 1 = 10; rat 2 = 17; rat 3 = 14; rat 4 = 12; and rat 5 = 8). The first epoch was excluded from decode-to-animal distance analysis as hippocampal place fields take around 5 min to stabilize in a new environment^67^. Each run session was interleaved with 15–20 min in an unrewarded rest box. Electrophysiological and video data were acquired using SpikeGadgets hardware and software (https://spikegadgets.com/trodes/, v.1.8.0). Running trajectories on the w-track were classified into outbound and inbound trials on different track regions, resulting in six different task phases during running: centre outbound; centre inbound; T-junction outbound; T-junction inbound; outer outbound; and outer inbound. Run periods for instantaneous speed and frequency analysis were defined using a velocity threshold of greater than 4 cm s^−1^, with a 250-ms buffer. Run periods for decode-to-animal distance and MUA trace modulation analysis were defined using a velocity threshold of greater than 10 cm s^−1^, with a 250-ms buffer.

### Behaviour tracking and monitoring of the stepping cycle

Underfloor video monitoring at 125 frames per second was performed using wide-angle rectilinear lenses (Theia Technologies; SL183M) mounted on AVT Manta cameras (AVT-GM-158C-POE-CS; per-frame exposure time: 7.5 ms) on both the transparent linear tracks and the w-tracks (abrasion-resistant polycarbonate sheets, TAP Plastics). To ensure that each camera frame was correctly assigned to a corresponding electrophysiological recording time, we captured both the neural data and the position data in a common reference frame using the precision time protocol (PTP). To aid limb identification, the forelimbs of the rats were painted with a white body paint (SportSafe) that contrasted with the black hoods of the Long-Evans rats. The hindlimbs were painted with black body paint to contrast with their white underbelly. A machine-learning algorithm, DeepLabCut^65^ (v.2.0.5.1), was trained to track the distinct body parts of the rats, including the nose, forelimbs, hindlimbs and base of the tail. The training dataset included frames from different track portions during various phases of the stepping cycle in both outbound and inbound trials. The model was allowed to run for the maximum number of iterations until its performance reached asymptote. The output comprises of *x*–*y* position coordinates for each labelled body part corresponding to each camera frame, along with a likelihood estimate. Position estimates with less than 0.99 likelihood were estimated as the interpolated value of the remaining estimates smoothed with a Gaussian window of 0.01 s. The velocity of the nose was smoothed with a Gaussian filter of 0.15 s (filters compensated for group delay). The same model was used to estimate position for all of the rats. For position analysis, the nose position was used as the actual position of the rat to correspond closely with previous work that uses an LED on the microdrive for tracking.

### Histology and recording-site assignment

In three rats, the left and right hippocampus was targeted at anteroposterior (AP): −4 mm, mediolateral (ML): ±2.6 mm; in one rat, the left and right hippocampus were targeted at AP: −3.8 mm and ML: ±2.6 mm; and in one rat only one hemisphere was targeted at AP: −3.72 mm and ML: +1.26 mm. A screw placed over the cerebellar cortex served as the global reference. Tetrode locations (four rats) were marked with electrolytic lesions after concluding the data acquisition. After a 24-h period to allow for gliosis, rats were perfused transcardially with 4% paraformaldehyde (PFA). The bottom of the brain was exposed, and the brain was left in 4% PFA overnight, after which the tetrodes were moved up, and the rest of the skull was removed. The brain was then transferred to a 30% sucrose solution for 5–7 days, sectioned into 50–100-μm slices and stored in 0.1 M phosphate-buffered saline with 0.02% (w/v) sodium azide. Sections were selected for Nissl staining to enable visualization of the locations of tetrode tips. Electrolytic lesion was not performed for one rat, but all subsequent steps were followed. We used the glial marker glial fibrillary acidic protein (GFAP) to localize these tetrodes.

### Data analysis

#### Statistical approach

A central goal of our analytical approach was to measure the synchronization between two variables—the stepping cycle and the hippocampal physiology. Each cycle of these rhythms provides us with a meaningful measurement of their synchronization. We then combine these measurements across individual passes through locations within an epoch, with the null hypothesis that the two rhythms are not correlated, and thus that they start at arbitrary phases relative to one another on each pass. We can compare these measurements to shuffled measurements (see ‘Shuffling analysis’) to derive a single value for each epoch that represents the tendency, across all cycles and passes within that epoch, for the two variables to be synchronized. Using this approach, we have computed all our metrics on every epoch and tested whether this synchronization is consistently present across the epochs within and across rats. Correspondingly, each statistical result is reported both across epochs within a rat and across all rats. Significance values were adjusted for multiple comparisons by the Benjamini–Hochberg method at a false discovery rate of 0.05.

#### Spike sorting

Hippocampal spikes were sorted using MountainSort (https://github.com/LorenFrankLab/franklab_mountainsort_old)^68^, an automatic clustering algorithm. The output of the algorithm is individual clusters with quality metrics. The quality metrics that were used to plot accepted clusters in Fig. 1a were the signal-to-noise ratio (>2), isolation score (>0.90), noise overlap (<0.3) and a visual inspection for refractory period violations. Note that sorted spikes were used only for the illustration of spiking activity in Fig. 1a.

#### Power spectral analysis

Power spectral analysis was performed during run periods using Welch’s method, and each segment was windowed with a Hamming window. The result is the power spectral density in each frequency bin (frequency resolution: 1 Hz) normalized by the maximum power observed at any bin per epoch. For calculating the peak frequency, we use a minimum peak height of 0.9.

#### Instantaneous frequency, speed and acceleration analysis

Stepping and theta data were filtered (stepping: each forelimb data was smoothed and bandpass-filtered between 1 Hz and 6 Hz with roll-offs at 0.5 and 8 Hz; theta: hippocampal theta data were bandpass-filtered between 6 Hz and 12 Hz with cut-offs at 4 Hz and 14 Hz using an acausal filter) and Hilbert-transformed, and their instantaneous frequency was computed by estimating the average phase difference at each time bin between windows of *t* *−* 125 ms and *t* + 125 ms. The instantaneous speed and acceleration were computed similarly in windows *t* *−* 125 ms and *t* + 125 ms as the mean of the observed values.

#### Clusterless decoding analysis

##### Inputs to the model

We created an encoding model that captured the associations between spike waveform features and the rat’s position at each 2-ms time bin as before^7^. The waveform feature used was the peak amplitude of each spike waveform on each of the four channels of the tetrode. Spikes were detected from the 600 Hz–6 kHz filtered signal when the amplitude on any channel of a tetrode exceeded a 100-μV threshold. The position of each rat was determined by converting the 2D position of the rat’s nose on the w-track to a 1D position on the basis of distance along the track segments (centre arm, outer arm and T-junction arm). This linearization is done to speed up the decoding. All trajectories begin with 0 cm representing the centre well position, and 15-cm gaps are placed between the centre arm, left arm and right arms in 1D space to prevent the smoothing across adjacent positions from influencing non-overlapping neighbouring segments inappropriately. The code used for linearization can be found at https://github.com/LorenFrankLab/track_linearization.

##### The model

We used a clusterless state space model (see ref. ^36^ for details) to decode the ‘mental position’ of the rat. Decoding used a 20-μV Gaussian smoothing kernel for the spike amplitude features and an 8-cm Gaussian smoothing kernel for position. The state space model had two movement dynamics—continuous and fragmented—which allowed the hippocampal representational trajectory of the rat to move both smoothly and discontinuously through space. This allows us to capture the full range of possible hippocampal spatial representations. The continuous dynamic was modelled by a random-walk transition matrix with a 6-cm standard deviation and the fragmented dynamic was modelled by a uniform transition matrix. The probability of staying in either the continuous or the fragmented movement dynamic was set to 0.968, which corresponds to 62.5 ms of staying in the same movement dynamic on average, or roughly the duration of half a theta cycle. We have shown that the model is relatively insensitive to this choice of parameter^7^. Decoding was done using a causal algorithm with uniform initial conditions for both movement dynamics. A 2-ms time bin and 2.5-cm position bin were used to allow for high-resolution decoding. We used fivefold cross validation for decoding, in which we encoded the relationship between waveform features and position on four-fifths of the data and then decoded the remaining fifth of the data. This ensures that the spikes that are used for constructing a given encoding model are not also used for decoding the representation. We repeated this for each fifth of the data.

##### Outputs of the model

Posterior probability of position: the posterior probability of position is a quantity that indicates the most probable ‘mental’ positions of the animal based on the data. We estimate it by marginalizing the joint probability over the dynamics.

Highest posterior density: the highest posterior density (HPD) is a measurement of the spread of the posterior probability at each time bin and is defined as the posterior region that contains the top 50% of the posterior probability values. Using the top values, this measurement of spread is not influenced by multimodal distributions (whereas an alternative measure like the quantiles of the distribution would be). In this manuscript, we use the HPD region size—the total area of the track covered by the 50% HPD region—to evaluate the uncertainty of the posterior probability of position.

Decode-to-animal distance: the distance between the decoded position and the actual position of the animal is defined as the shortest path distance between the most likely decoded position (the maximum of the posterior probability of position) and the animal’s position at each 2-ms time bin. The shortest path distance was calculated using Dijikstra’s algorithm^69^ on a graph representation of the track, in which the most likely decoded position and the rat’s position were inserted as nodes on this graph.

##### Epoch inclusion criteria for decode-to-animal distance analysis

For analysing the modulation of the decode-to-animal distance trace around forelimb plant times, we included only those epochs in which we could reliably decode the position across multiple inbound and outbound runs. We estimated this by evaluating a decode quality metric as follows. First, for every run, we computed the mean of the highest posterior density values and the mean of the absolute distance of the decoded position from the current position of the rat. We labelled runs in which either of these values exceeded 50 cm to be ‘noisy’; that is, cases in which we could not reliably estimate the position of the rat. We then defined the decode noise metric (ranging from 0–1) as the proportion of the length of noisy data to the length of all the data. Those epochs in which the decode noise metric was less than 0.25 for each arm of the w-track, and in which the rat ran each arm at least 10 times, were included in the analysis.

#### Forelimb plant times

The absolute difference of position data was calculated to obtain the instantaneous velocity of each forelimb (that is, the stepping cycle; one value per camera frame). This stepping cycle was then low-pass-filtered to 6 Hz with a roll-off at 8 Hz to remove outliers and noise events. The stance and swing portions of the stepping cycle correspond to the times when the acceleration of the limb is the minimum and the maximum, respectively. An acceleration profile for each limb was created to identify peaks and troughs of stepping rhythm, which was used to define the start and end times of the stance and swing phases. Plant times were defined as the midpoint of 10–30% of the stance phase, and lift times were defined as the midpoint of 10–30% of the swing phase. These times correspond to the limbs of the rat fully touching or not touching the track’s surface. These plant times were validated with data from a rat running on a transparent track, in which we also used a 45-degree mirror to obtain the side view. We then manually annotated camera frames (blind to the plant times) when the individual fingers of a reference forelimb splayed fully, indicating starting load on the forelimb (‘fingersplay’ times, from the bottom track view), and camera frames when the reference forelimb first touched the surface of the track completely (‘touchdown’ times from the side mirror view). We then compared these manually annotated times to the ‘plant times’ detected by our algorithm (above) and found a close correspondence between these times (plant–fingersplay median offset, IQR = 0.008s, 0.016 s; number of plants = 114; plant–touchdown median offset, IQR = 0.008s, 0.008s; number of plants = 66; Extended Data Fig. 1 and Supplementary Video 2).

#### Step-representational content coupling

To measure the coupling of steps and the content of hippocampal representations, we first identified the peaks of the decode-to-animal-distance trace (minimum peak height 10 cm) during the outbound runs on the centre arm on the track, and then computed the represented position in windows of ±10 ms around the detected peak using the peak of the posterior in that time window. Thus, each such non-local representational instance was assigned to representing the centre (0), right (1) or left (−1) arm. Then, for each assigned non-local representation, we determined whether the preceding forelimb plant was from the right (1) or left (−1) forelimb. To ask whether there is a consistent organization between the parity of step and the content of internal hippocampal representation (for example, left plant followed by right representation, and vice versa or left plant followed by left representation, and so on), we included those runs that had at least two instances of non-local representation. Then we computed the proportion of runs in which we saw a step-representation alternation (that is, left plant followed by right representation, and right plant followed by left representation) and the proportion of runs in which we saw a step-representation correspondence (that is, left plant followed by left representation and right arm followed by right representation).

#### MUA

For detecting MUA events, a histogram of spike counts was constructed using 1.5-ms bins; all spikes greater than 100 μV on tetrodes in the CA1 cell layer were included. The MUA trace was smoothed with a Gaussian kernel (15-ms standard deviation).

#### Decode-to-animal distance and MUA modulation score

First, we calculated the forelimb-plant-triggered average of the decode-to-animal distance or MUA trace for each epoch in a time window of ±70 ms. Then, we computed the modulation score by calculating the sum of absolute deviations from the mean of the observed values in the decode-to-animal distance or MUA-triggered trace per epoch. To compare these raw modulation scores across task phases and epochs, we *z*-scored them using the mean and standard deviation obtained from the null distribution (description below) matched for observed forelimb plants per epoch per rat. All observed plants were included for MUA analysis. For the analysis of the decode-to-animal distance, we included only those sequences that engaged a mental exploration further ahead of the current position of the rat by at least 10 cm (ref. ^39^). Then, each forelimb plant was evaluated in a window of ±50 ms, and the goodness of the decode-to-animal distance trace was computed in this window by calculating the number of time bins with a highest posterior density greater than 50 cm. If these values exceeded a total of 10 ms, then those plants were excluded from analysis as we could not reliably estimate the structure of the decoded position adjacent to those plants.

#### Shuffling analysis

Plant times were randomly offset between −70 ms and 70 ms, 5,000 times, keeping the inter-event times intact. An event-triggered average of these shuffled times was computed to create the superset of the shuffled distribution data. Then a matched number of events (plants) as observed in the data were randomly selected 1,000 times per epoch to create a null distribution of shuffled modulation scores.

#### Quantification and statistical analysis

All analyses were performed using custom code written in MATLAB v.2020a (Mathworks) and Python v.3.6. Statistical tests used and significance values are provided throughout the text and in figure legends.

### Reporting summary

Further information on research design is available in the Nature Portfolio Reporting Summary linked to this article.

## Online content

Any methods, additional references, Nature Portfolio reporting summaries, source data, extended data, supplementary information, acknowledgements, peer review information; details of author contributions and competing interests; and statements of data and code availability are available at 10.1038/s41586-023-05928-6.

## Supplementary information

Reporting Summary

Supplementary Video 1 Transparent track running trajectory and DeepLabCut limb tracking. The undertrack video of animal running on linear track showing consistent tracking of the nose, tail, and limbs of the rat during movement. Video begins at 1x speed and then repeats at 0.1x speed.

Supplementary Video 2 Validation of the method for detecting forelimb plant times. Undertrack video of a rat running on a transparent track, with a 45-degree mirror to additionally visualize the side view of the forelimb during one complete gait cycle. The video is slowed down 50 times (0.02x).

Supplementary Video 3 Video summary of step-hippocampal representation relationship. Forelimb plant times are synchronized with hippocampal neural representations. The undertrack video captures the beginning of an outbound trial where the animal traverses the centre arm of the track toward the choice point and then turns to its left. Note that the w-track is oriented such that the reward wells are on the left side of the video frame. The green dot represents the decoded position (peak of the posterior density) estimated from the firing of hippocampal neurons. The video is slowed down 32 times and pauses briefly at each detected forelimb plant time as the animal runs towards the spatial decision point on the centre arm. The right forelimb plants are shown in purple, and the left forelimb plants are shown in orange. The video is flipped so that the animal moving towards the outer arm at the top of the video frame corresponds to a left well/left turn in the real world.

## Data Availability

All data used for this study are publicly available in the DANDI Archive at https://dandiarchive.org/dandiset/000410/draft/. Any additional information required to reanalyse the data reported in this paper is available from the corresponding authors upon request.

## Code availability

All code used for the analysis of this data is publicly available at https://github.com/LorenFrankLab and https://zenodo.org/deposit/7615939.
