# Supplementary material for: Dynamic synchronization between hippocampal representations and stepping
Source: Nature. 2023 Apr 12;617(7959):125–31. doi: 10.1038/s41586-023-05928-6 (PMC10156593; doi:10.1038/s41586-023-05928-6)
Supplement: Supplementary file 1 — Reporting Summary [file 41586_2023_5928_MOESM1_ESM.pdf]

nature portfolio

Corresponding author(s): Abhilasha Joshi and Loren Frank

Last updated by author(s): Feb 9, 2023

Reporting Summary

Nature Portfolio wishes to improve the reproducibility of the work that we publish. This form provides structure for consistency and transparency in reporting. For further information on Nature Portfolio policies, see our Editorial Policies and the Editorial Policy Checklist.

Statistics

For all statistical analyses, confirm that the following items are present in the figure legend, table legend, main text, or Methods section.

n/a	Confirmed	
☐	☒	The exact sample size (*n*) for each experimental group/condition, given as a discrete number and unit of measurement
☐	☒	A statement on whether measurements were taken from distinct samples or whether the same sample was measured repeatedly
☐	☒	The statistical test(s) used AND whether they are one- or two-sided<br>*Only common tests should be described solely by name; describe more complex techniques in the Methods section.*
☒	☐	A description of all covariates tested
☐	☒	A description of any assumptions or corrections, such as tests of normality and adjustment for multiple comparisons
☐	☒	A full description of the statistical parameters including central tendency (e.g. means) or other basic estimates (e.g. regression coefficient) AND variation (e.g. standard deviation) or associated estimates of uncertainty (e.g. confidence intervals)
☐	☒	For null hypothesis testing, the test statistic (e.g. F, t, r) with confidence intervals, effect sizes, degrees of freedom and P value noted<br>*Give P values as exact values whenever suitable.*
☐	☒	For Bayesian analysis, information on the choice of priors and Markov chain Monte Carlo settings
☒	☐	For hierarchical and complex designs, identification of the appropriate level for tests and full reporting of outcomes
☐	☒	Estimates of effect sizes (e.g. Cohen's *d*, Pearson's *r*), indicating how they were calculated

Our web collection on statistics for biologists contains articles on many of the points above.

Software and code

Policy information about availability of computer code

Data collection	Electrophysiological and video data were acquired using Spike Gadgets hardware and software (https://spikegadgets.com/trodes/). We used Trodes version 1.8.2 to collect, and extract the data used in this study.
Data analysis	All analyses were performed using custom code written in MATLAB 2020a (Mathworks) and Python3.6. Statistical tests used and significance values are provided throughout the text and in figure legends. Each analysis was done per epoch and the summary results are analyzed both per animal and for all animals combined. We also report adjusted p values calculated using the Benjamini-Hochberg method throughout the manuscript.

For manuscripts utilizing custom algorithms or software that are central to the research but not yet described in published literature, software must be made available to editors and reviewers. We strongly encourage code deposition in a community repository (e.g. GitHub). See the Nature Portfolio guidelines for submitting code & software for further information.

Data

Policy information about availability of data

All manuscripts must include a data availability statement. This statement should provide the following information, where applicable:

- Accession codes, unique identifiers, or web links for publicly available datasets
- A description of any restrictions on data availability
- For clinical datasets or third party data, please ensure that the statement adheres to our policy

All data used for this study are publicly available in the DANDI Archive at https://dandiarchive.org/dandiset/000410/draft/. All code used for the analysis of this data is publicly available at https://zenodo.org/deposit/7615939. Any additional information required to reanalyze the data reported in this paper is available from the corresponding author/s upon request.

Field-specific reporting

Please select the one below that is the best fit for your research. If you are not sure, read the appropriate sections before making your selection.

☒ Life sciences ☐ Behavioural & social sciences ☐ Ecological, evolutionary & environmental sciences

For a reference copy of the document with all sections, see nature.com/documents/nr-reporting-summary-flat.pdf

Life sciences study design

All studies must disclose on these points even when the disclosure is negative.

Sample size	Individual rats were treated as replicates and all metrics are computed per animal. The total number of rats included in this study is similar to other rat electrophysiology studies. e.g. 10.1016/j.neuron.2021.07.029 and 10.1523/JNEUROSCI.2614-14.2015
Data exclusions	Those epochs/times where excluded where we could not reliably estimate position decoded
Replication	We have computed all our metrics on every epoch and tested the hypothesis whether this synchronization is consistently present across the epochs within and across animals. Correspondingly, each statistical result is reported both across epochs within an animal and across all animals.
Randomization	We used randomization for computing the step-representation and step-mua synchronization. The modulation score for the observed data in each case was compared to the 95% CI bounds of a shuffled distribution described in methods.
Blinding	We did not have separate behavioral cohorts as we needed real time information of behavioral performance from these high-density electro-physiological recordings. The analysis was then split by task-phase in the w-track task (Outbound and Inbound) providing comparison periods for the strength of synchronization observed.

Reporting for specific materials, systems and methods

We require information from authors about some types of materials, experimental systems and methods used in many studies. Here, indicate whether each material, system or method listed is relevant to your study. If you are not sure if a list item applies to your research, read the appropriate section before selecting a response.

Materials & experimental systems

n/a	Involved in the study	
☒	☐	Antibodies
☒	☐	Eukaryotic cell lines
☒	☐	Palaeontology and archaeology
☐	☒	Animals and other organisms
☒	☐	Human research participants
☒	☐	Clinical data
☒	☐	Dual use research of concern

Methods

n/a	Involved in the study	
☒	☐	ChIP-seq
☒	☐	Flow cytometry
☒	☐	MRI-based neuroimaging

Animals and other organisms

Policy information about studies involving animals; ARRIVE guidelines recommended for reporting animal research

Laboratory animals	Rattus norvegicus; Long Evans; Males; 5-9 months old
Wild animals	No wild animals were used in this study
Field-collected samples	No field collected samples were used in this study
Ethics oversight	All experimental procedures were in accordance with the University of California San Francisco Institutional Animal Care and Use Committee and US National Institutes of Health guidelines.

Note that full information on the approval of the study protocol must also be provided in the manuscript.
